# Severe Presentation of Nontypeable *Haemophilus influenzae* (NTHi) Infection in a Previously Healthy Toddler

**DOI:** 10.1155/2019/8306491

**Published:** 2019-09-15

**Authors:** Eiman Al Boloushi, Abdulla Al Amri, Ghassan Ghatasheh, Huda Al Dhanhani

**Affiliations:** ^1^Academic Affair Department, Tawam Hospital, Al Ain, UAE; ^2^General Pediatrics Department, Tawam Hospital, Al Ain, UAE; ^3^Pediatrics Infectious Disease Department, Tawam Hospital, Al Ain, UAE

## Abstract

**Background:**

Cellulitis is the inflammation of the skin and subcutaneous tissue. It is usually caused by Gram-positive organisms such as *Staphylococcus and Streptococcus pyogenes* infection. Nontypeable *Haemophilus influenzae* (NTHi) is an uncommon cause of cellulitis. Hence, we report on this case.

**Case Presentation:**

A previously healthy 19-month-old girl presented with a fever and two-day history of progressive right leg swelling and redness. Her physical examination revealed significant induration and swelling of her right lower leg but no obvious signs of abscess formation. Given the clinical picture, she was admitted as a case of cellulitis. Parenteral clindamycin was started empirically, as the blood culture preliminary report showed Gram-negative rods. Ceftriaxone was added to broaden the coverage. Final blood culture grew NTHi. Despite the use of proper antibiotics (amoxicillin clavulanic acid), the clinical course was complicated with abscess formation that required surgical intervention.

**Conclusions:**

We are reporting a previously healthy child who developed NTHi cellulitis of the lower leg. To the best of our knowledge, there have been no formal reports pertaining to leg cellulitis following infection by NTHi, yet published in UAE, and reports of HIB cellulitis of the extremities still appear to be rare; hence, we report on this case.

## 1. Introduction

*Haemophilus influenzae* is a Gram-negative coccobacillus bacterium. There are two major types of *Haemophilus* species, the typeable *Haemophilus influenzae* and nontypeable *Haemophilus influenzae* (NTHi), which were classified according to the availability of the polysaccharide layer in its capsule [1]. *Haemophilus influenzae* can cause multiple infectious diseases such as meningitis, pneumonia, septicemia, epiglottitis, septic arthritis, osteomyelitis, cellulitis, and peritonitis, and it is also one of the HACEK organisms which contribute to infective endocarditis (HACEK: *Haemophilus*, *Actinobacillus*, *Cardiobacterium*, *Eikenella*, and *Kingella*) [2]. Cellulitis due to *Haemophilus* species is very rare to be encountered in the clinical practice.

We are reporting a case of severe presentation of nontypeable *Haemophilus influenzae* skin and soft tissue infection in a previously healthy toddler, an unusual clinical presentation with diagnostic and treatment challenges.

## 2. Case Summary

A previously healthy 19-month-old girl presented with a two-day history of right-leg progressive edema and erythema associated with high fever, irritability, and reduced appetite. She was limping and unable to bear weight due to pain. The patient had a recent history of insect bites in multiple areas of her body including both legs. She was fully immunized including HIB (*Haemophilus influenza* type B) vaccination as per the United Arab Emirates childhood vaccination schedule. The family history was unremarkable. Her 3 siblings were well, and there was no family history of consanguinity or immunodeficiency.

On examination, the patient had numerous marks of insect bites all over the body, particularly the exposed skin areas. There was significant induration and swelling of her right lower leg but no obvious signs of abscess formation. Otherwise, she looked systemically stable with normal vital signs, and her cardiovascular and respiratory examinations were normal. She had no hepatosplenomegaly.

**Figure 1:**
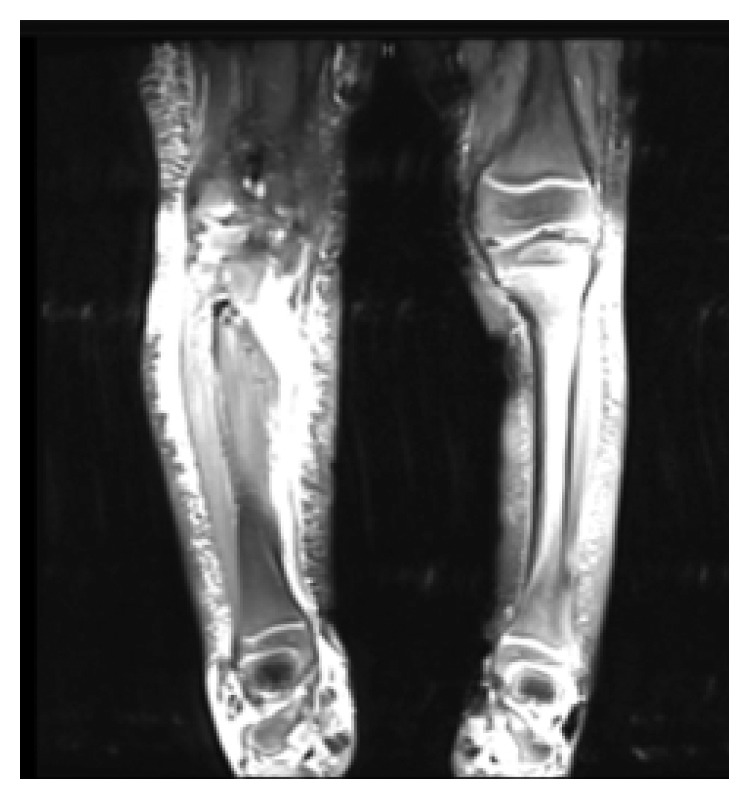
MRI scab showing the deep tissue inflammation and soft tissue edema.

Given the clinical picture, the girl was admitted with a provisional diagnosis of cellulitis. She underwent a partial septic screen, which showed an increased total white cell count (WCC 20.2 × 10^9^/L), significantly elevated C-reactive protein (CRP 348.5 mg/L), and anemia for age (Hb 106 g/L). Her platelet count was 348 × 10^9^/L. Blood cultures were obtained, and she was started on parenteral clindamycin. The blood culture was reported to be positive in one bottle for Gram-negative rods after 18 hours of incubation; therefore, a third generation cephalosporin (ceftriaxone) was added to broaden antimicrobial cover. Final identification revealed nontypeable *Haemophilus influenzae* bacteria where serotype was not performed, as it is not available. Despite the broad-spectrum antibiotics, the girl continued to suffer high-grade fever and progressive swelling in her right leg, despite repeatedly negative blood cultures. Furthermore, she developed bilateral purulent conjunctivitis, which was thought to be caused by NTHi. She underwent MRI imaging (Figure 1) and an ultrasound scan to exclude deep tissue collection or bone/joint involvement. The imaging showed evidence of soft tissue edema and signs of inflammation with free fluid between the muscles but no signs of osteomyelitis or septic arthritis.

**Figure 2:**
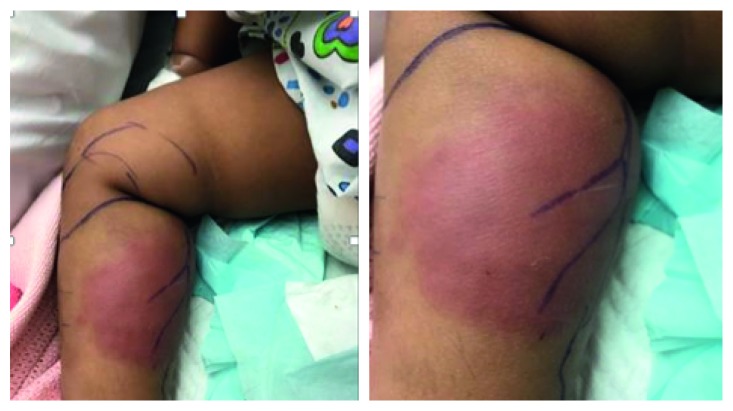
Right leg swelling and redness.

The possibility of staphylococcal infection (MSSA, methicillin-sensitive *S. aureus*) was also suspected; therefore, ceftriaxone was switched to Augmentin (amoxicillin and clavulanate). The patient made clinical improvement; however, after 8 days, she developed localized erythema and swelling (Figure 2). The repeated ultrasound scan at this point showed a significant fluid collection (5 cm × 10 cm) requiring incision and drainage. During the procedure, she was noted to have marked fat tissue necrosis, which raised the possibility of necrotizing fasciitis. On the other hand, the histological examination of the collected specimens showed findings consistent with abscess formation with no evidence of acute necrotizing fasciitis with negative culture. Following the surgical intervention, the patient improved dramatically with no further fevers and settling inflammatory markers, and she was able to walk again with normal gait. She had a repeated MRI scan, which showed no evidence of osteomyelitis. Given the severity of complicated cellulitis caused by the unusual organism, the girl underwent immunological assessment and investigations including lymphocyte subsets and complement studies, which were completely normal.

## 3. Discussion

The patient described above suffered from invasive skin and soft tissue infection due to *Haemophilus influenzae* which is a Gram-negative coccobacillus discovered in 1889. The bacterium is described to be a nonmotile, non-spore-forming organism and has the ability to grow in different environmental conditions (aerobically and anaerobically). There are multiple serotypes of typeable strains which were further classified according to polysaccharides in their capsule into A, B, C, D, and F, while *Haemophilus influenzae* type B (HIB) is the most common strain isolated from infections in the clinical practice [1]. The dramatic decline in the invasive disease due to HIB since the introduction of HIB conjugate vaccines has been well documented [3, 4]. In our case with invasive NTHi disease, the most striking observation is the young age. This observation is supported by the studies from the United Kingdom. Falla et al. identified 24 children in Oxford, England, with serious NTHi disease during the period from 1985 to 1991 and found that 83% of these children were less than 3 years old [5], and it was also reported by CDC that the global incidence of invasive infections related to NTHi was 1.3 per 100,000 in children younger than 5 years [6]. The predominance of disease among young children may reflect the high prevalence of nasopharyngeal colonization with NTHi and the high incidence of viral respiratory infection (and therefore increased mucosal inflammation and the potential for bacterial invasion) in this age group [7]. It has also been reported that levels of immunoglobulin (IgG, IgM, and IgA) antibodies to NTHi are relatively low until 2 years of age, reaching adult levels by 4 years of age [8]. In contrary to other reported risk factors for developing invasive NTHi [9, 10], our patient did not have underlying medical problems, but it might be related to high virulence strain of *Haemophilus* as the bacteria has multiple virulence factors, such as the ability to produce specific enzymes that can cleave protective immunoglobulin A (IgA) that protects the mucosal layer, which is called the bacterial IgA protease enzyme. A recent study by Vitovski et al. revealed that invasive isolates of NTHi exhibit increased IgA1 protease activity compared to isolates from asymptomatic carriers [11]. The level of IgA1 protease activity was not assayed in our case, but it may be one factor that contributes to the patients with NTHi invasive disease. Moreover, the *Haemophilus* bacterium can also survive in the respiratory epithelial cells which is called intracellular sequestration, which might explain not only its colonization in the respiratory tract epithelium but also that it can migrate below the cells through transmural migration to the subepithelial area and get access to the vascular system, resulting in severe invasive infections like bacteremia and meningitis [4]. Another factor described by Arnold et al. is the ability of NTHi to form microcolonies on its mucosal surfaces which can inhibit the function of the secreted bacteriostatic products such as lactoferrin and lysozymes and to some potential can inhibit the antibody production [12].

It is known in the literature that the protection against invasive *H. influenzae* requires both innate and acquired humoral immunity. Similar to other infections, maternal antibodies against HIB were noticed to protect newborns against this strain, but they are still prone to infections with nontypeable strains. Furthermore, breastfeeding provides some protection against *H. influenzae* [13]. Although the associated clinical manifestations in NTHi infections make it a target for vaccine development, there are some challenges, e.g., the lack of the capsular polysaccharides and the described high rate of heterogeneity among the isolates from the same infection site [14]. In our patient, we thought about the possibility of primary immune deficiency contributing to the severe presentation since the patient was previously immunized, but no immune defect was identified as she had normal complete blood count, normal immunoglobulin level, and lymphocyte subset.

In conclusion, skin and soft tissue infection due to nontypeable *Haemophilus influenzae* species is quite rare, and there are few case reports in the literature; therefore, we suggest keeping it in consideration in patients with immune deficiency and those who responds slowly to the empirical antimicrobials with limited coverage. Moreover, we described in our patient slow response to the nonsurgical approach, and therefore, we encourage early surgical intervention in cases of complicated *Haemophilus influenzae* cellulitis with collections. On the other hand, more research is needed to understand risk factors for such invasive infection.
